# Like a bridge over troubled water – a qualitative study of professional caregiver singing and music as a way to enable person-centred care for persons with dementia

**DOI:** 10.1080/17482631.2020.1735092

**Published:** 2020-03-26

**Authors:** Anna Swall, Lena Marmstål Hammar, Åsa Gransjön Craftman

**Affiliations:** a Department of Nursing Science, Sophiahemmet University, Stockholm, Sweden; b School of Health, Care and Social Welfare, Mälardalen University, Västerås, Sweden; c School of Education, Health and Social Studies, Dalarna University, Falun, Sweden; d Division of Nursing, Department of Neurobiology, Care Sciences, Karolinska Institutet, Stockholm, Sweden

**Keywords:** Caregiver singing, dementia care, person-centred care, communication, music, qualitative method

## Abstract

**Purpose**: To describe the perspectives of caregivers in terms of using singing and music in their everyday work, and of their effect on care and interaction with the person with dementia.

**Methods**: A qualitative design was used, consisting of group discussions with professional caregivers from three nursing homes in a medium-sized city in a rural area of Sweden.

**Results**: The results demonstrate that caregiver singing and music can be powerful and useful in the care of and in communication with persons with dementia. Music, for example, can be used to facilitate socialization as it opens up for discussion, while caregiver singing was preferable when it came to the facilitation of care situations and interaction.

**Conclusions**: Singing and music can be powerful and useful tools in the care of and in communication with persons with dementia. Regardless of whether singing or music is used, the most important factor is that a person-centred approach is adopted so as to make the music a facilitative tool. Caregiver singing and music are ways to connect with the person with dementia and an understanding of their use can contribute to dementia research. This in turn can increase awareness of the possible ways to strengthen the partnership between caregivers and persons with dementia.

## Introduction

The older population is increasing worldwide, the result being a growing number of PWDs. This presents a global challenge since PWDs require comprehensive healthcare services (Association, 2016; WHO, 2010). PWDs suffer from major cognitive impairment, and so-called behavioural and psychological symptoms of dementia (BPSDs) are common. Person-centred interventions are preferred as ways to prevent or handle situations that occur because of BPSDs (McConnell & Karel, 2016; Sahin Cankurtaran, 2014). Singing and music as interventions are widely used and have positive effects on mood, communication and cooperation between PWDs and caregivers, thus facilitating care situations and decreasing BPSDs (Hammar Marmstål, 2013; Hammar Marmstål, Emami, Engstrom, & Gotell, 2010; Pedersen, Andersen, Lugo, Andreassen, & Sutterlin, 2017). However, to be able to use them in a structured way, more information is needed on how singing and music affect the care of PWDs and their encounters with caregivers. This knowledge is valuable since it can provide a more careful description of how to use singing and music strategically in the care of PWDs, and furthermore this can be a component in the education of caregivers.

## Background

The term Behavioural and Psychological Symptoms of Dementia (BPSDs) refers to behaviour such as screaming, wandering, resisting care and acting aggressively, both verbally and physically (Hugo & Ganguli, 2014). More than 80% of PWDs develop BPSDs as their disease progresses (Geda et al., 2013; Ismail et al., 2016). BPSDs are especially common in PWDs’ interaction with others and when the personal integrity of the PWDs is threatened, such as during care situations (Miyamoto, Tachimori, & Ito, 2010; Wang et al., 2016). This is challenging for both the caregivers as well as PWDs. To minimize BPSDs, pharmacological treatments are available. However, these medications are associated with strong side effects, including an increased risk for falls and fractures, as well as strokes (Berry et al., 2016). In addition, they may be associated with an increased risk of mortality (Gardette et al., 2012), and what is more the relief they provide is only temporary (Cerejeira, Lagarto, & Mukaetova-Ladinska, 2012; Gardette et al., 2012). Risks with anti-psychotic medication outweigh its benefits in terms of BPSDs, and as such non-pharmacological treatments should be considered first (Dyer, Harrison, Laver, Whitehead, & Crotty, 2018; Gardette et al., 2012; Kales, Gitlin, & Lyketsos, 2015; Seitz et al., 2012; Wang et al., 2016). Non-pharmacological treatments are cost-effective but are underused despite the fact that they can potentially reduce BPSDs without the risks associated with pharmacological treatment (Gerlach & Kales, 2018; Herrmann & Gauthier, 2008; Scales, Zimmerman, & Miller, 2018). To be successful, the caring of PWDs should be person-centred (Du Toit, Shen, & McGrath, 2018; Fazio, Pace, Flinner, & Kallmyer, 2018; McNiel & Westphal, 2018), meaning that care should be individualized and based on the fact that every PWD is unique. Further, care should focus on meaningfulness: that is to say, the care should involve activities that are meaningful to the PWD (Downs & Lord, 2017; McCance, McCormack, & Dewing, 2011). Such activities may involve music, such as music therapy.

### Music in PWD care

Musical activities have been used in dementia care for decades, and a Cochrane review (van der Steen et al., 2017) focusing on musical activities and music therapy showed positive results when it came to reducing some BPSDs, such as depression. In a meta-analysis by (Pedersen et al., 2017), agitation was shown to decrease in music therapy sessions. The making of music, such as singing together in groups, has been evaluated by Evans, Garabedian and Bray (2017), and results demonstrate that the engagement and positive social interaction of PWDs increased during such activities. Similar results were found by Camic, Williams, and Meeten (2013) in studies related to when PWDs sang together with family members who were their caregivers. However, van der Steen et al. (2017) suggest that further studies should focus on long-term effects and positive outcomes, such as well-being and positive emotion. In addition, since most BPSDs manifest themselves during care situations involving interaction with caregivers (Wang et al., 2016), interventions that facilitate these are priority.

### Caregiver singing in dementia care

Singing in care situations showed itself in a study by Sarkamo, Altenmuller, Rodriguez-Fornells, and Peretz (2016) to be energizing, refreshing and stress-reducing for both PWDs and caregivers. In residential care, singing in care situations, so-called Caregiver Singing or Music Therapeutic Caregiving (MTC), was defined as being “when the caregivers are singing for or together with persons with dementia during caring”: this was first described by Götell, Brown, and Ekman (2000). Several studies focused on this in morning care situations and demonstrate that even though the songs were not about the caring activity, communication and cooperation with the PWD and the caregiver increased, which facilitated care situations (Dennis, 2011; Götell, Brown, & Ekman, 2003; Hammar Marmstål et al., 2010). In addition, BPSDs, referred to as resistance to care, aggression and screaming, decreased while positive emotions, such as smiling, laughing and singing, increased (Dennis, 2011; Götell et al., 2003; Götell, Brown, & Ekman, 2009; Hammar Marmstål, Emami, Engström, & Götell, 2010; Hammar Marmstål, Emami, Gotell, & Engstrom, 2011). Also, caregivers describe themselves as being more relaxed and happier while singing (Hammar Marmstål, Emami, Engstrom, & Götell, 2011). During mealtimes, caregiver singing increased communication, and the PWDs were more relaxed and more eager to eat (Hammar Marmstål, 2013; Hammar Marmstål, Williams, Swall, & Engström, 2012).

Because mutual engagement and interaction with PWDs and their caregivers are commonly problematic, interventions that focus on facilitation are important. Studies on caregiver singing show promising results. However, they have thus far focused on specialized structured care situations (morning care and mealtimes). As caregiver singing can be used in almost all situations, because the caregivers use their own voice as an instrument, studies need to focus on when it is preferable to sing, why caregivers decide to sing and how they use singing. Focus also needs to be on the effects for both the PWDs and the caregivers. Furthermore, music is widely used in different forms: caregiver singing is one way, and is commonly complemented by music-making, background music or conversations about music. This makes it problematic to draw conclusions in terms of when it is preferable to use music rather than caregiver singing, and vice versa. In addition, previous studies have mainly focused on the successful use of music and singing, with little being noted about when its use has negative outcomes. Thus, studies need to focus on both how and when music and/or caregiver singing can be useful in interactions with PWDs and their caregivers, and how and when they are not useful. Results of such studies are valuable since they can serve to form a needed structure and basis in the education of caregivers that includes the use of caregiver singing and music. As a step towards developing this structure, this study aims to describe the caregivers’ perspectives on using caregiver singing and music in their everyday work, and how singing and music influences care and interaction with PWDs.

## Method

The study has a qualitative approach with the model of World Café discussions (Brown & Isaacs, 2001) as the data collection method. This method enhances collaborative thinking, with group work serving as a way to share knowledge about a particular subject. Content analysis (Elo & Kyngäs, 2008) was chosen for the analysis of the group discussions.

### Context and participants

The study was conducted in a medium-sized city in a rural area of Sweden. Caregivers working at three nursing homes for PWDs in the city were offered training in how to use music in different ways in their everyday work. In our aim to recruit study participants, the second author contacted the managers of nursing homes to request permission to carry out the study at their nursing homes, and this they gave. The caregivers at the nursing homes who attended the training about music and caregiver singing were also informed orally about this study. After that, they received an envelope containing information about the study and a form that they were asked to complete with regards to their willingness to participate in the study. This they were then asked to send by post to the second author of the study. Of 37 caregivers at the lecture, 30 from the three nursing homes signed the consent form and thus agreed to participate in the study.

The nursing homes each had approximately 60 PWD residents, with wards that accommodated about ten PWDs. In each ward, there were between six and eight caregivers, and some of these took part in this study. Of the 30 participants, one was a man and the rest women. They were aged between 24 and 63, with a mean age of 50. Twenty-six were trained assistant nurses, and the four others were nurse’s aides. They had been working within healthcare for between two and 42 years, with a mean of 22.8, and within dementia care between two and 36 years, with a mean of 15.1. Inclusion criteria were that they must have attended a lecture (which were included in the training) on caregiver singing and music (described below) and must have practiced caregiver singing and music in the care of the PWDs they worked with.

### Intervention and data collection

As part of the training, the caregivers participated in a four-hour lecture given by the second author that was about theories on the influence of music on humans and on the use of music and caregiver singing in dementia care. The lecture included instructions on how to choose songs based on person-centredness as well as examples based on previous studies of when it could be preferable for caregivers to use music or caregivers singing. After the lecture, the caregivers were asked to use music and caregiver singing with one or several PWDs at the nursing home where they worked. They themselves decided on the situations in which they preferred to use them based on what they knew about the PWDs. After two months, they were invited to participate in the data collection for this study. The data collection consisted of group discussions structured in such a way as to focus on the use of caregiver singing and music and on the outcomes, with focus on both the feelings and behaviour of the PWDs and the caregivers themselves, as well as on the situations in which they used either caregiversinging or music—or indeed both. With the aim to have participants relax when telling their stories and also to have open discussions on the subject, these discussions were structured according to the World Café Model (Brown & Isaacs, 2001). This model provides open conversations about a pre-decided subject and is structured using a semi-structured conversation guide based on the aim of the study. The groups consisted of five to eight participants who sat around a table at one of the nursing homes to discuss the questions (Table I). They were audio-recorded upon their permission. The discussions lasted from 32 to 56 minutes. The audio files were transcribed verbatim.

**Table I.** Group discussion questions about care for persons with dementia

How did the person with dementia express himself before you began using caregiver singing? What are your reasons for introducing songs into your care practice? For example: Communication? BPSD?
How did the person with dementia express himself during the caregiving? For example: communication? BPSD?
How did you feel when you used caregiver singing? Positively and/or negatively?
What is the most common motivational reason for using caregiver singing? BPSD? Special situation?
How do persons with dementia react? Positively and/or negatively?
When does it not work? What happens then? Are there special situations?
In which situations do you prefer to use singing?
In which situations do you prefer to use music?

### Data analysis

An inductive qualitative content analysis, described by Elos and Kyngäs (2008), was conducted of the data, and all interviews were analysed as a whole unit. Initially, the text was read repeatedly to identify the content. Subsequently, meaningful units (meaning units or sentences) relating to the aim of the study were identified and labelled with a code related to their content. The meaning units and the codes were then transferred to a matrix. Thereafter, depending on similar descriptions or statements in the meaning units and the codes, they were interpreted, abstracted and grouped into subcategories named with content-characteristic words. Each subcategory was analysed with regards to similarities and differences, and was grouped into generic categories, which by the end formed one main category based on similarities. Each step in the analysis process was characterized by flexibility and repetitive verification of the original text, which was discussed within the research group. Any discrepancies in the analyses were examined until consensus within the research group was reached. All members of the research group had experience conducting research in this field. An example of the analysis process is shown in Table II.

**Table II.** Example of the analysis process

Meaning unit	Condensed meaning unit Description close to the text	Sub-category	Generic category	Main category
We have a woman on our ward. And when she sits down, she refuses to stand up again. You cannot pull her up from the chair, but if you sing and do some dance moves and invite her to dance, she stands up willingly.	A woman who refuses to rise from a chair but responds by standing up when you sing and invite her to dance.	Caregiver singing and music promote mutual communication.	Caregiver singing and music are tools to promote interaction with persons with dementia.	Caregiver singing and music build bridges towards person-centred care.
Then I can tell you about a situation with a man. We sat and listened to Elvis (Presley) and after a little while he started to sing and finally he took hold of my hand, and yes you know, you get the feeling, he was so moved. So it is clear that music can mean a lot. Absolutely.	One man became very emotional when we listened to Elvis (Presley). He started to sing and held my hand. It touched me and it is clear that music can mean a lot.	Caregiver singing and music give rise to emotional expressions.	Caregiver singing and music bring out a glimpse of the person.	

In this study (Table III), the two generic categories *Caregiver singing and music are tools to promote interaction with persons with dementia* and *Caregiver singing and music bring out a glimpse of the person* formed the main category: *Caregiver singing and music build bridges towards person-centredness*.

**Table III.** Sub-category, Generic category, Main category

Sub-category	Generic category	Main category
Caregiver singing and music promote mutual communication	Caregiver singing and music are tools to promote interaction with persons with dementia	Caregiver singing and music build bridges towards person-centredness
Caregiver singing and music facilitate the caregiving encounter		
Caregiver singing and music give rise to emotional expressions	Caregiver singing and music bring out a glimpse of the person	
Caregiver singing and music awake dormant abilities		

## Research ethics and patient consent

The study was approved by The Swedish Ethical Review Authority and was allocated the register number 2016/507. In line with the Declaration of Helsinki (2018), participants were informed both orally and in writing by the third author about the aim of the study and research ethics: for example, the voluntary nature of participation. All participants signed a written consent form with regards to their participation in the study. The researcher and participants did not know each other. The PWDs mentioned in the study were unknown to the authors.

## Results

The findings resulted in one overarching main category ‘Caregiver singing and music build bridges towards person-centredness’, which in turn was developed into two generic categories and four sub-categories (Table III).

## Caregiver singing and music build bridges towards person-centredness

Caregiver singing and music are useful tools to build bridges in a person-centred way when it comes to connecting with PWDs. They were mostly valuable, joyful and meaningful, but needed to be person-centred and needed to be adapted according to the state of health of the PWDs at the time. They were a way to promote togetherness that in turn facilitated socialization and cooperation between PWDs and caregivers.

### Caregiver singing and music are tools to promote interaction with persons with dementia

Informants explained how singing and music could promote communication when speaking was no longer useful in communication with the PWD. Singing and music become respectful means to reach out to PWDs.

#### Caregiver singing and music promote mutual communication

they were described as an opportunity to frame reciprocal communication, sometimes even without words, such as in a dance or while listening to music together. This was a moment of closeness with PWDs in which feelings of relaxation or joy were apparent. The caregivers especially described singing as being a way to communicate in a playful manner.

I usually sing when I walk into his bedroom: good morning, good morning now shines the sun. The PWD used to laugh oh, oh, are you here again? (G2)

The caregivers described caregiver singing as a bridge that facilitated what the caregiver expected the PWD to do. For example, requesting a PWD to rise through song was seen as a more gentler approach than a spoken instruction or command to ”stand up”. Singing seemed easier for PWDs, who may have lost the ability to interpret spoken instructions, to understand. In such a way, caregiver singing was a useful form of communication.

We had a lady in our ward, and she was …, she never wanted to get out of bed … we knew that she liked music, so I came in and sang one time, and she started singing with me. She sang along very well … I thought it was great fun myself. (G6)

Singing together in the caring situation, for example when walking in the corridor, was described as helpful for PWDs, who seemed to have an easier time concentrating and walking. The caregiver chose to sing a song that would motivate PWDs to move rather than to give a command that might not affect the situation.

We have a lady who refuses to get up from a chair. Once she has sat down, you cannot pull or drag her up from the chair. But then I sing and do some dance moves, and then she stands up with no problem. (G1)

The caregivers stated that singing, humming and music have always been seen as natural elements in the care of PWDs. Caregivers describe how singing as a means to communicate can make both the situation and the day more comprehensible for PWDs.

#### Caregiver singing and music bring out a glimpse of the person

they facilitate the caregiving encounter, and this had a positive effect on caregivers. They were able to interact more positively with PWDs using caregiver singing and music, making for a more pleasant and easier situation.

I can also tell you about a gentleman who was listening to Elvis. So we sat down together, and he started singing and he took my hand … both of us were moved by the situation. So it is clear that singing can mean a lot for every human. (G1)

If the caregivers liked music or sang along to music on the radio, either alone or together with PWDs, they described a sense of light-heartedness and *“something nice happens to you and your mood when you sing.”*

It has a really positive effect, singing. It is fun. It is more fun to work then, and then maybe the person hears that you are singing, that you are happy as well, and then the mood changes (G6).

They also described how pleasant it was to hear colleagues sing in caring situations to ease the mood, and it did not matter how or what was being sung. These situations had an impact on the caregivers’ mood, which in turn affected the caring situation and contributed to a positive atmosphere throughout the day.

Younger male PWDs were often described as having different tastes in music than the older PWDs in the wards. As such, it was common for younger male caregivers to assist the younger male PWDs. Often, the younger PWDs liked hard rock bands, such as Iron Maiden or Kiss. Their taste in music and bands was seen as valuable and a source of mutual interest in something, and a discussion between the PWDs and the caregivers as fans of the bands could develop. This affected the care situation in a positive way, and as such, caregivers were commonly paired with PWDs according to their taste in music, at least as far as was possible.

### Caregiver singing and music bring out a glimpse of the person

Caregiver singing and music were described to be emotional and to appeal to personal experiences.

#### Caregiver singing and music give rise to emotional expressions

this describes how music is a personal matter since it affects feelings, mood and memories; however, how the music is received by PWDs varies.

One woman I cared for would absolutely not listen to any music when she didn’t feel very good and was sad. She only wanted to talk about the past as far back as she could remember, yet, when she was happy, she wanted to listen to music. (G3)

Knowing the life stories of PWDs and choosing the right music for the person could bring back memories and open up for conversations about life and the way PWDs recall life. However, their taste in music was described as changing, possibly as a result of the stage of dementia they had.

You may want to listen to a completely different kind of music today than you did in your earlier life. Previously, one old man listened to Ted Gärdestad, and he sang every time he heard the songs. But now he doesn’t react at all when we put them on. (G6)

The caregivers also stated how a previous preference of a PWD may become uninteresting or even a source of negative feelings. Moreover, the music that was once preferred may lose its significance. One example was given of a woman who had been active in the Salvation Army but who now showed no interest in the psalms, and as such, the caregivers perceived that to be a closed chapter in the woman’s life.

… this woman had listened to religious music all her life, but when she moved here, she did not want to listen to it anymore. (G5)

However, caregivers described how some PWDs had clear memories of the music they had enjoyed or songs they had sung in their earlier lives and associated these with activities, and that these memories and the emotions they evoked were preserved, regardless of their dementia.

One lady who was a hundred years old and who had severe dementia watched a church service on TV. She was very moved by the psalms she heard, and suddenly asked me to get money from her purse so that she could put it in the collection box. (G1)

Music was viewed to be meaningful in several ways: it draws people to gather around the TV to watch a musical, and listening to music opens up for talk about the music, artists, actors and eras from the lives of PWDs. Music could bring about a wide range of emotions, including stress, and if played at the wrong time could invoke feelings of aggression and irritation, or perhaps even worsen such emotions.

Singing songs that had special meaning to PWDs could arouse emotions. Mostly the singing appeared to be comforting. Two caregivers describe their experiences as follows:

1- But it can also be so emotional. I played the song, you know, from the movie about the poor children. I will paint the whole world for you little mother. It became so emotional, we just hugged.

2- I also care for a woman who bursts into tears every time I sing (G1)

Singing was not always suitable in the care of some PWDs. One example given is of a PWD who became very distressed during toileting situations and showering, and expressed this by screaming and refusing to sit down. The woman usually enjoyed singing and music, but not in this situation. Caregivers described the importance of observing carefully the reactions and emotions of the PWD so as to determine the appropriateness of caregiver singing and music and preferences in terms of which music to play. Two caregivers described their experiences with PWDs when it came to music choice:

1- You can see from her face how she really enjoys these nice songs. I guess they bring back memories and the lyrics are beautiful.3 -None of ours (PWDs) react in that way (facial expressions). (G4)

The caregivers found it sometimes hard to foresee the effect of their choice of song before the harm was done. This was sometimes the case when a childhood song was being sung—one that the PWD knew well—but in the situation it was wrong. It was important not to make the PWD feel like a child in the situation.

#### Singing and music awake dormant abilities

this refers to the fact that it was satisfying to see when a PWD reacted to music even in a small way, like simply moving a finger or foot to the beat of the music. The caregivers also described dance evenings as being very popular. The music played was itself described as entertaining and amusing for the PWD, but with dancing as well, the experience was even more valuable and meaningful, and could result in unexpected physical movements.

2- I will never forget the man from your ward [at a dance evening], the one who sat in a wheelchair and could not connect or communicate in any way, he also had to be fed. He sat there in the wheelchair and suddenly you could see him moving his feet! It was amazing. (G4)

It was common for the caregivers to be asked to dance by PWDs, who could demonstrate that they were competent dance partners and that they were able to follow the music.

1—We (the PWDs and the caregiver) stood in here and listened to the live music, then we went up and danced.- 2 Wow1- He liked dancing, and then you really could see what the music could do for him, from being just tired and frozen and then he gave it his all. It almost made me cry. (G3)

The caregivers also explained how age and health were irrelevant: singing and music were seen as a way to reach out and activate PWDs.

## Discussion

The study aimed to describe caregivers’ perspectives on using caregiver singing and music in their everyday work, and how these influenced the care and the interaction with the PWDs.

Results show that caregiver singing and music can be used to promote interaction between caregivers and PWDs; they can also be seen as a way to bring out a glimpse of the person´s innermost feelings. Listening to music or watching a musical facilitated socialization as they opened up for discussions, while caregiver singing was preferable in caring situations and interactions. This is valuable knowledge when it comes to the development of education programmes for caregivers, as previous research has commonly focused on singing and music together, and not on their specific uniqueness. Moreover, according to this study, caregiver singing and music facilitated mutual interaction between the PWD and the caregiver, which is recognized as crucial in person-centred dementia care (Edvardsson, 2008). This is because the caregivers were able to identify and acknowledge the needs of the PWDs, and were able to comfort them in their sadness and share moments of joy that came with listening to music or singing. Evans, Garabedian, and Bray (2019) have also recognized the stress-reducing effect of singing in care situations. Evans et al (2017) demonstrated how PWDs and caregivers can sit down and watch a familiar musical as a social activity, just as our study also demonstrates. These activities were seen as memory recallers and served as an opportunity to socialize and talk about both the music and the actors in the musical.

In this study, the caregivers, based on their experience, stated that caregiver singing was a gentle way to guide the PWDs instead of instructing them during care situations. The PWDs were responsive, for instance, to rising from a chair when focusing on the singing and being invited to dance. Their willingness to interact and to be aware in the present moment of caregiver singing is also described in (Hammar Marmstål, 2011, 2013; Hammar Marmstål et al., 2010). Our findings on how caregiver singing can be used instead of talking and how it facilitated the encounter was also found in previous studies on caregiver singing, as mentioned above. This is also shown in research on PWD communication and speech (Fu, Belza, Nguyen, Logsdon, & Demorest, 2018; Sihvonen et al., 2017) that showed that singing may be preferable in certain situations and phases of dementia instead of regular speech in which words may have lost their meaning. Stewart, von Kriegstein, Warren, and Griffiths (2006) have studied this and explain that music activates several parts of the brain, depending on many factors such as rhythm, melody, accent and timbre, and they suggest that the combination of language and music, as with singing, offers a greater chance of activating intact neurological pathways other than simply speech.

Another important aspect for our study is the choice of song or music in terms of person-centred care (Edvardsson, Winblad, & Sandman, 2008; Kitwood, 1997). The songs and music must be chosen based on the preferences of the individual PWD if they are to have a positive effect. Personalized music has shown itself in previous studies to improve speech (Dassa & Amir, 2014), and the memories and emotions of PWDs connect to music that is personalized (Dassa & Amir, 2014; Ridder, Stige, Qvale, & Gold, 2013; Spiro, 2010). Negative emotions, such as aggression, could also be evoked, as demonstrated in our study. In previous research, there is a duality (Pedersen et al., 2017; Steen van der et al., 2017) regarding the effects of agitation and aggression, a fact shown by this study. Some PWDs could open up with the singing and music, while some became even more stressed and agitated. With this in mind, it may be more important to be clear about which music and songs each person likes. As such, the life story of the PWD may be very valuable. However, our results show that songs that PWDs have liked earlier in their lives may no longer be interesting and may even evoke negative emotions. As such, this leaves the caregivers with the task of figuring out which music to use in their PWD encounters so as to increase the chance of their forming a good relationship. As described in our study, the songs and music chosen were sometimes unknown to the caregivers; further, it was not only the playing of music or singing that seemed to be significant to PWDs, but also the talking about music. Some caregivers had problems using music with those PWDs who did not know the music. One example is the younger PWDs who favoured hard rock music—for example, Iron Maiden and Kiss. This requires a deep understanding for a person-centred approach as these PWDs might have other needs than those of the older PWDs. McCormack (2003) describes person-centredness as the possibility to arrive at decisions that are truly the person’s own, decisions that express everything that the person believes to be important about him-/herself. The flexibility that the caregivers need to have so as to meet the needs of the younger PWDs in care (for example, they need to consider music preferences) places high demands on the caregivers’ experience and knowledge about person-centred care. This was also confirmed by Downs and Lord (2017), and McCance et al. (2011).

The caregivers also found that singing made them joyful and less stressed, which is another aspect of this study. This confirms findings from a previous study by Hammar Marmstål et al. (2011). This might have to do with the less pronounced BPSDs of the PWDs, as well as the fact that PWDs became more responsive, and communicated and interacted in a positive manner (Evans et al., 2017); it could also be because the music enabled them as humans to connect to their emotions and to each other (Cespedes-Guevara & Eerola, 2018; Reybrouck, Eerola, & Podlipniak, 2018). Wang and Agius (2018) suggest that music can have positive effects on humans, and that music making and in particular singing can serve to help humans relax and increase their sense of positivity, which could be related to our study for both PWDs and the caregiver. Our results also showed an increase in engagement and communication, and Koelsch (2014) explains this by suggesting that music serves to trigger engagement in social activities; further, it is well recognized that socialization is a basic human need that involves communication, interaction and a sense of social belonging.

In summary, the findings of our study show that the caregiver and the PWD can connect with each other through caregiver singing, and this allows bridges to be built and an unspoken understanding of the situation. They meet at a level that only singing makes possible, and this allows for a person-centred connection. This is valuable knowledge when it comes to the development of education programmes for caregivers, as previous research has commonly focused on singing and music together, and not on their specific uniqueness.

## Strengths and limitations

Data for the study was collected through group discussions (Brown & Isaacs, 2001), this format being viewed as an appropriate method of data collection for several reasons.

Group discussions that do not have a researcher leading them, as was the case in our study, allow for social interaction, and this makes it easier for participants to describe and explore a specific research area without the presence of a researcher. Instead, the pre-designed topic guide enhanced the study’s credibility and gave participants an opportunity to discuss and share their experiences of caregiver singing and music. This approach was positive in terms of eliciting a broad range of data. Our impressions are such that group discussions allowed for an open climate for reflection and that they stimulated dialogue and the exchange of experiences. However, one limitation with this data collection method is that it did not allow for probing questions as is the case with individual or focus group interviews. Such interviews would probably have allowed for more detailed descriptions of caregiver experiences and clearer descriptions of when to use music or caregiver singing: such descriptions were not as detailed as we would have hoped for. It should also be noted that the second author gave the lecture on music and caregiver singing, and this may have led to the participants may have felt obliged to participate in the study. However, this was not our impression as the discussions were lively and that participants did not hold back on talking about their experiences; further, they described both the positive and negative sides of music and caregiver singing.

The choice in terms of qualitative content analysis was made according to the characteristics of the data. The data was descriptive probably because of the limitation of not being able to pose probing questions or to steer the group discussions, In a future study, we plan to use focus group interviews or individual interviews conducted by a researcher as a means to collect data that will allow for an indepth analysis. In this study, qualitative content analysis was useful for structuring the text and allowed us to move back and forth between the whole text and parts of it, which increased trustworthiness. Trustworthiness also increased as a result of the analysis conducted in cooperation with the research group. The analysis process was discussed continuously in the research group so that consensus could be reached. There was a clear and detailed clarification of the analysis process so that dependability could be established. Furthermore, the quotations in the section on findings also contribute to the reliability of this study. More than a few of the findings were consistent with other studies in the field, and different views on the subject have been described in the results so as to promote transferability to other settings, such as in home care services and/or in informal care for PWDs.

## Conclusion and implications

Our conclusion is that caregiver singing and music can be useful in different situations: caregiver singing to facilitate care situations and music to increase socialization. Regardless of whether it is singing or music that is used, what is of upmost importance is a person-centred approach. We further conclude that caregivers need to be responsive to the preferred music of younger PWDs and related activities since they probably have other views and needs than older PWDs who normally receive care in nursing homes for PWDs. Future studies should also focus on younger PWDs and the role of music and caregiver singing in thecare specified for them.

With reference to both our study and previous studies, music and caregiver singing should be seen as useful tools in the care of PWDs in challenging situations—that is to say, not only as an enjoyable activity. It can be argued that training in this area should figure in education programmes, especially when the focus is on older persons and dementia. However, caregivers in elderly care and in dementia care commonly lack appropriate education, while financial resources in this sector are frequently low. As such, education related to music and caregiver singing from an organizational perspective is difficult to prioritize when there are other, arguably more pressing, issues to consider. In addition, knowledge about the benefits of caregiver singing and music are generally low on management level and is commonly seen as a pleasant activity instead of an intervention that the organization would benefit from. Further research is needed on long-term structured interventions for the different PWD sub groups. As well, future studies should focus on caregiver singing and music that examine the differences between the two in a healthcare setting.
